# On solving split mixed equilibrium problems and fixed point problems of hybrid-type multivalued mappings in Hilbert spaces

**DOI:** 10.1186/s13660-017-1416-x

**Published:** 2017-06-15

**Authors:** Nawitcha Onjai-uea, Withun Phuengrattana

**Affiliations:** 1 Department of Mathematics, Faculty of Science and Technology, Nakhon Pathom Rajabhat University, Nakhon Pathom, 73000 Thailand; 2 Research Center for Pure and Applied Mathematics, Research and Development Institute, Nakhon Pathom Rajabhat University, Nakhon Pathom, 73000 Thailand

**Keywords:** 47N10, 47J20, 47J22, 46N10, split mixed equilibrium problems, hybrid multivalued mappings, weak convergence, Hilbert spaces

## Abstract

In this paper, we introduce and study iterative algorithms for solving split mixed equilibrium problems and fixed point problems of *λ*-hybrid multivalued mappings in real Hilbert spaces and prove that the proposed iterative algorithm converges weakly to a common solution of the considered problems. We also provide an example to illustrate the convergence behavior of the proposed iteration process.

## Introduction

Let *H* be a real Hilbert space with inner product $\langle\cdot,\cdot\rangle$ and induced norm $\Vert \cdot \Vert $. Let *C* be a nonempty closed convex subset of *H*, $\varphi:C\rightarrow \mathbb{R}$ be a function, and $F: C\times C\rightarrow\mathbb{R}$ be a bifunction. The *mixed equilibrium problem* is to find $x\in C$ such that

1.1 $$ F(x,y)+\varphi(y)-\varphi(x)\geq0, \quad \forall y \in C. $$

The solution set of mixed equilibrium problem is denoted by $\mathit{MEP}(F,\varphi)$. In particular, if $\varphi=0$, this problem reduces to the equilibrium problem, which is to find $x\in C$ such that $F(x,y)\geq0, \forall y \in C$. The solution set of equilibrium problem is denoted by *EP(F)*.

The mixed equilibrium problem is very general in the sense that it includes, as special cases, optimization problems, variational inequality problems, minimization problems, fixed point problems, Nash equilibrium problems in noncooperative games, and others; see, *e.g.*, [1–4].

In 1994, Censor and Elfving [5] firstly introduced the following split feasibility problem in finite-dimensional Hilbert spaces: Let $H_{1}$, $H_{2}$ be two Hilbert spaces and *C*, *Q* be nonempty closed convex subsets of $H_{1}$ and $H_{2}$, respectively, and let $A:H_{1}\rightarrow H_{2}$ be a bounded linear operator. The split feasibility problem is formulated as finding a point $x^{*}$ with the property

$$ x^{*}\in C \quad\text{and}\quad Ax^{*}\in Q. $$

The split feasibility problem can extensively be applied in fields such as intensity-modulated radiation therapy, signal processing and image reconstruction, then the split feasibility problem has received so much attention by so many scholars; see [6–9].

In 2013, Kazmi and Rizvi [10] introduced and studied the following split equilibrium problem: let $C\subseteq H_{1}$ and $Q\subseteq H_{2}$. Let $F_{1}:C\times C\rightarrow\mathbb{R}$ and $F_{2}:Q\times Q\rightarrow\mathbb{R}$ be nonlinear bifunctions and let $A:H_{1}\rightarrow H_{2}$ be a bounded linear operator. The *split equilibrium problem* is to find $x^{*}\in C$ such that

1.2 $$ F_{1} \bigl(x^{*},x \bigr)\geq0,\quad \forall x\in C \text{ and such that } y^{*}=Ax^{*}\in Q \text{ solves } F_{2} \bigl(y^{*},y \bigr)\geq0, \forall y\in Q. $$

The solution set of the split equilibrium problem is denoted by

$$\mathit{SEP}(F_{1},F_{2}):= \bigl\{ x^{*}\in C: x^{*}\in \mathit{EP}(F_{1}) \text{ and } Ax^{*}\in \mathit{EP}(F_{2}) \bigr\} . $$

The authors gave an iterative algorithm to find the common element of sets of solution of the split equilibrium problem and hierarchical fixed point problem; for more details refer to [11, 12].

In 2016, Suantai *et al.* [13] proposed the iterative algorithm to solve the problems for finding a common elements the set of solution of the split equilibrium problem and the fixed point of a nonspreading multivalued mapping in Hilbert space, given sequence $\{x_{n}\}$ by

1.3 $$\begin{aligned} \textstyle\begin{cases} x_{1}\in C \quad\text{arbitrarily}, \\ u_{n}=T^{F_{1}}_{r_{n}} (I-\gamma A^{*} (I-T^{F_{2}}_{r_{n}} )A )x_{n}, \\ x_{n+1}\in\alpha_{n} x_{n}+(1- \alpha_{n})Su_{n}, \quad\forall n\in \mathbb{N}, \end{cases}\displaystyle \end{aligned}$$

where $\{\alpha_{n}\} \subset(0,1)$, $r_{n}\subset(0,\infty)$ and $\gamma\in(0,\frac{1}{L})$ such that *L* is the spectral radius of $A^{*}A$ and $A^{*}$ is the adjoint of *A*, $C\subset H_{1}$, $Q\subset H_{2}$, $S:C\rightarrow K(C)$ is a $\frac{1}{2}$-nonspreading multivalued mapping, $F_{1}:C\times C\rightarrow\mathbb{R}$ and $F_{2}:Q\times Q\rightarrow\mathbb{R}$ are two bifunctions. The authors showed that under certain conditions, the sequence $\{x_{n}\}$ converges weakly to an element of $F(S)\cap \mathit{SEP}(F_{1},F_{2})$.

Several iterative algorithms have been developed for solving split feasibility problems and related split equilibrium problems; see, *e.g.*, [14–16].

Motivated and inspired by the above results and related literature, we propose an iterative algorithm for finding a common element of the set of solutions of split mixed equilibrium problems and the set of fixed points of *λ*-hybrid multivalued mappings in real Hilbert spaces. Then we prove some weak convergence theorems which extend and improve the corresponding results of Kazmi and Rizvi [10] and Suantai *et al.* [13] and many others. We finally provide numerical examples for supporting our main result.

## Preliminaries

Let *C* be a nonempty closed convex subset of a real Hilbert space *H*. We denote the strong convergence and the weak convergence of the sequence $\{x_{n}\}$ to a point $x\in H$ by $x_{n}\longrightarrow x$ and $x_{n}\rightharpoonup x$, respectively. It is also well known [17] that a Hilbert space *H* satisfies *Opial’s condition*, that is, for any sequence $\{x_{n}\}$ with $x_{n}\rightharpoonup x$, the inequality

$$\limsup_{n\longrightarrow \infty} \Vert x_{n}-x \Vert < \limsup _{n\longrightarrow \infty} \Vert x_{n}-y \Vert $$

holds for every $y\in H$ with $y\ne x$.

The following two lemmas are useful for our main results.

### Lemma 2.1

*In a real Hilbert space* *H*, *the following inequalities hold*:

1. $\Vert x-y \Vert ^{2} \leq \Vert x \Vert ^{2}- \Vert y \Vert ^{2} - 2\langle x-y,y\rangle, \forall x,y\in H$;
2. $\Vert x+y \Vert ^{2} \leq \Vert x \Vert ^{2} + 2\langle y, x + y\rangle, \forall x,y\in H$;
3. $\Vert t x+(1-t)y \Vert ^{2} = t \Vert x \Vert ^{2}+(1-t) \Vert y \Vert ^{2}-t (1-t) \Vert x-y \Vert ^{2}, \forall t \in[0,1], \forall x,y\in H$;
4. *If* $\{x_{n}\}$ *is a sequence in* *H* *which converges weakly to* $z\in H$, *then*
  $$\limsup_{n\rightarrow\infty} \Vert x_{n}-y \Vert ^{2}= \limsup_{n\rightarrow\infty} \Vert x_{n}-z \Vert ^{2}+ \Vert z-y \Vert ^{2}, \quad\forall y\in H. $$

### Lemma 2.2

[18]

*Let* *H* *be a Hilbert space and* $\{x_{n}\}$ *be a sequence in* *H*. *Let* $u,v\in H$ *be such that* $\lim_{n\rightarrow\infty} \Vert x_{n}-u \Vert $ *and* $\lim_{n\rightarrow\infty} \Vert x_{n}-v \Vert $ *exist*. *If* $\{x_{n_{k}}\}$ *and* $\{x_{m_{k}}\}$ *are subsequences of* $\{x_{n}\}$ *which converge weakly to* *u* *and* *v*, *respectively*, *then* $u=v$.

A single-valued mapping $T : C\longrightarrow H$ is called *δ-inverse strongly monotone* [19] if there exists a positive real number *δ* such that

$$\begin{aligned} \langle x-y,Tx-Ty\rangle\geq\delta \Vert Tx-Ty \Vert ^{2}, \quad \forall x,y\in C. \end{aligned}$$

For each $\gamma\in(0,2\delta]$, we see that $I-\gamma T$ is a nonexpansive single-valued mapping, that is,

$$\bigl\Vert (I-\gamma T)x-(I-\gamma T)y \bigr\Vert \leq \Vert x-y \Vert ,\quad \forall x,y\in C. $$

We denote by $CB(C)$ and $K(C)$ the collection of all nonempty closed bounded subsets and nonempty compact subsets of *C*, respectively. The *Hausdorff metric* $\mathcal{H}$ on $CB(C)$ is defined by

$$ \mathcal{H}(A,B):=\max \Bigl\{ \sup_{x\in A} \operatorname{dist} (x,B), \sup_{y\in B} \operatorname{dist} (y,A) \Bigr\} , \quad \forall A,B \in CB(C), $$

where $\operatorname{dist}(x,B)=\inf\{d(x,y):y\in B\}$ is the distance from a point *x* to a subset *B*. Let $S:C\rightarrow CB(C)$ be a multivalued mapping. An element $x\in C$ is called a *fixed point* of *S* if $x\in Sx$. The set of all fixed points of *S* is denoted by $F(S)$, that is, $F(S)=\{x\in C:x\in Sx\}$. Recall that a multivalued mapping $S:C\rightarrow CB(C)$ is called

(i) *nonexpansive* if
  $$ \mathcal{H}(Sx,Sy)\leq \Vert x-y \Vert ,\quad x,y\in C; $$
(ii) *quasi-nonexpansive* if $F(S)\neq\emptyset$ and
  $$ \mathcal{H}(Sx,Sp)\leq \Vert x-p \Vert , \quad\forall x\in C, \forall p \in F(S); $$
(iii) *nonspreading* [13] if
  $$ 2\mathcal{H}(Sx,Sy)^{2}\leq \operatorname{dist}(y,Sx)^{2}+ \operatorname{dist}(x,Sy)^{2},\quad \forall x,y\in C; $$
(iv) *λ-hybrid* [20] if there exists $\lambda\in \mathbb{R}$ such that
  $$ (1+\lambda)\mathcal{H}(Sx,Sp)^{2}\leq(1-\lambda) \Vert x-y \Vert ^{2}+\lambda \operatorname{dist}(y,Sx)^{2}+\lambda \operatorname{dist}(x,Sy)^{2},\quad \forall x,y\in C. $$

We note that 0-hybrid is nonexpansive, 1-hybrid is nonspreading, and if *S* is *λ*-hybrid with $F(S)\ne\emptyset$, then *S* is quasi-nonexpansive. It is well known [20] that if *S* is *λ*-hybrid, then $F(S)$ is closed. In addition, if *S* satisfies the condition: $Sp=\{p\}$ for all $p\in F(S)$, then $F(S)$ is also convex.

The following result is a demiclosedness principle for *λ*-hybrid multivalued mapping in a real Hilbert space.

### Lemma 2.3

[20]

*Let* *C* *be a nonempty closed convex subset of a real Hilbert space* *H* *and* $S:C\rightarrow K(C)$ *be a* *λ*-*hybrid multivalued mapping*. *If* $\{x_{n}\}$ *is a sequence in* *C* *such that* $x_{n}\rightharpoonup x$ *and* $y_{n}\in Sx_{n}$ *with* $x_{n}-y_{n} \rightarrow 0$, *then* $x\in Sx$.

For solving the mixed equilibrium problem, we assume that the bifunction $F_{1}:C\times C \rightarrow\mathbb{R}$ satisfies the following assumption:

### Assumption 2.4

Let *C* be a nonempty closed and convex subset of a Hilbert space $H_{1}$. Let $F_{1}:C\times C \rightarrow\mathbb{R}$ be the bifunction, $\varphi:C\rightarrow\mathbb{R}\cup\{+\infty\}$ is convex and lower semicontinuous satisfies the following conditions:

1. $F_{1}(x,x)=0$ for all $x\in C$;
2. $F_{1}$ is monotone, *i.e.*, $F_{1}(x,y)+F_{1}(y,x)\leq 0, \forall x,y \in C$;
3. for each $x,y,z \in C$, $\lim_{t\downarrow 0}F_{1}(tz+(1-t)x,y)\leq F_{1}(x,y)$;
4. for each $x\in C$, $y\mapsto F_{1}(x,y)$ is convex and lower semicontinuous;
5. for each $x\in H_{1}$ and fixed $r>0$, there exist a bounded subset $D_{x}\subseteq C$ and $y_{x}\in C$ such that, for any $z\in C\setminus D_{x}$,
  $$ F_{1}(z,y_{x})+\varphi(y_{x})- \varphi(z)+\frac{1}{r}\langle y_{x}-z,z-x\rangle< 0; $$
6. C is a bounded set.

### Lemma 2.5

[21]

*Let* *C* *be a nonempty closed and convex subset of a Hilbert space* $H_{1}$. *Let* $F_{1}:C\times C\rightarrow \mathbb{R}$ *be a bifunction satisfies Assumption* 2.4 *and let* $\varphi:C\rightarrow\mathbb{R}\cup\{+\infty\}$ *be a proper lower semicontinuous and convex function such that* $C\cap \operatorname{dom} \varphi\neq\emptyset$. *For* $r>0$ *and* $x \in H_{1}$. *Define a mapping* $T^{F_{1}}_{r}:H_{1}\rightarrow C$ *as follows*:

$$T^{F_{1}}_{r}(x)= \biggl\{ z\in C: F_{1}(z,y)+ \varphi(y)- \varphi(z)+\frac{1}{r}\langle y-z,z-x\rangle\geq0, \forall y \in C \biggr\} , $$

*for all* $x\in H_{1}$. *Assume that either* (B1) *or* (B2) *holds*. *Then the following conclusions hold*:

1. *for each* $x\in H_{1}$, $T^{F_{1}}_{r}\neq\emptyset$;
2. $T^{F_{1}}_{r}$ *is single*-*valued*;
3. $T^{F_{1}}_{r}$ *is firmly nonexpansive*, *i*.*e*., *for any* $x,y\in H_{1}$,
  $$\bigl\Vert T^{F_{1}}_{r}x-T^{F_{1}}_{r}y \bigr\Vert ^{2}\leq \bigl\langle T^{F_{1}}_{r}x-T^{F_{1}}_{r}y,x-y \bigr\rangle ; $$
4. $F(T^{F_{1}}_{r})=\mathit{MEP}(F_{1},\varphi)$;
5. $\mathit{MEP}(F_{1},\varphi)$ *is closed and convex*.

*Further*, *assume that* $F_{2}:Q\times Q\rightarrow\mathbb{R}$ *satisfying Assumption* 2.4 *and* $\phi:Q\rightarrow \mathbb{R}\cup\{+\infty\}$ *is a proper lower semicontinuous and convex function such that* $Q\cap \operatorname{dom} \phi\neq\emptyset$, *where* *Q* *is a nonempty closed and convex subset of a Hilbert space* $H_{2}$. *For each* $s>0$ *and* $w\in H_{2}$, *define a mapping* $T^{F_{2}}_{s}:H_{2}\rightarrow Q$ *as follows*:

$$T^{F_{2}}_{s}(v)= \biggl\{ w\in Q: F_{2}(w,d)+\phi(d)- \phi(w)+\frac {1}{r}\langle d-w,w-v\rangle\geq0, \forall d\in Q \biggr\} . $$

*Then we have the following*:

(6) *for each* $v\in H_{2}$, $T^{F_{2}}_{s}\neq\emptyset$;
(7) $T^{F_{2}}_{s}$ *is single*-*valued*;
(8) $T^{F_{2}}_{s}$ *is firmly nonexpansive*;
(9) $F(T^{F_{2}}_{s})=\mathit{MEP}(F_{2},\phi)$;
(10) $\mathit{MEP}(F_{2},\phi)$ *is closed and convex*.

## Main results

In this section, we prove the weak convergence theorems for finding a common element of the set of solutions of split mixed equilibrium problems and the set of fixed points of *λ*-hybrid multivalued mappings in real Hilbert spaces and give a numerical example to support our main result.

We introduce the definition of split mixed equilibrium problems in real Hilbert spaces as follows.

### Definition 3.1

Let *C* be a nonempty closed convex subset of a real Hilbert space $H_{1}$ and *Q* be a nonempty closed convex subset of a real Hilbert space $H_{2}$. Let $F_{1}:C\times C\rightarrow\mathbb{R}$ and $F_{2}:Q\times Q\rightarrow\mathbb{R}$ be nonlinear bifunctions, let $\varphi:C\rightarrow\mathbb{R}\cup\{+\infty\}$ and $\phi:Q\rightarrow\mathbb{R}\cup\{+\infty\}$ be proper lower semicontinuous and convex functions such that $C\cap \operatorname{dom}\varphi \neq\emptyset$ and $Q\cap \operatorname{dom}\phi\neq\emptyset$, and let $A:H_{1}\rightarrow H_{2}$ be a bounded linear operator. The *split mixed equilibrium problem* is to find $x^{*}\in C$ such that

3.1 $$\begin{aligned} F_{1} \bigl(x^{*},x \bigr)+\varphi(x)-\varphi \bigl(x^{*} \bigr) \geq0, \quad \forall x\in C, \end{aligned}$$

and such that

3.2 $$\begin{aligned} y^{*}=Ax^{*}\in Q \quad \text{solves}\quad F_{2} \bigl(y^{*},y \bigr)+ \phi(y)-\phi \bigl(y^{*} \bigr)\geq 0, \quad \forall y\in Q. \end{aligned}$$

The solution set of the split mixed equilibrium problem (3.1) and (3.2) is denoted by

$$\mathit{SMEP}(F_{1},\varphi,F_{2},\phi):= \bigl\{ x^{*}\in C: x^{*}\in \mathit{MEP}(F_{1},\varphi) \text{ and } Ax^{*}\in \mathit{MEP}(F_{2},\phi) \bigr\} . $$

We now get our main result.

### Theorem 3.2

*Let* *C* *be a nonempty closed convex subset of a real Hilbert space* $H_{1}$ *and* *Q* *be a nonempty closed convex subset of a real Hilbert space* $H_{2}$. *Let* $A: H_{1}\rightarrow H_{2}$ *be a bounded linear operator and* $S:C\rightarrow K(C)$ *a* *λ*-*hybrid multivalued mapping*. *Let* $F_{1}:C\times C\rightarrow\mathbb{R}$, $F_{2}:Q\times Q\rightarrow\mathbb{R}$ *be bifunctions satisfying Assumption* 2.4, *let* $\varphi:C\rightarrow\mathbb{R}\cup\{+\infty\}$ *and* $\phi:Q\rightarrow\mathbb{R}\cup\{+\infty\}$ *be a proper lower semicontinuous and convex functions such that* $C\cap \operatorname{dom} \varphi \neq\emptyset$ *and* $Q\cap \operatorname{dom} \phi\neq\emptyset$, *respectively*, *and* $F_{2}$ *is upper semicontinuous in the first argument*. *Assume that* $\Theta=F(S)\cap \mathit{SMEP}(F_{1},\varphi,F_{2},\phi) \neq\emptyset$ *and* $Sp=\{p\}$ *for all* $p\in F(S)$. *Let* $\{x_{n}\}$ *be a sequence generated by* $x_{1}\in C$ *and*

3.3 $$\begin{aligned} \textstyle\begin{cases} u_{n}=T^{F_{1}}_{r_{n}} (I-\gamma A^{*} (I-T^{F_{2}}_{r_{n}} )A )x_{n}, \\ y_{n}=\alpha_{n}x_{n}+(1-\alpha_{n})w_{n},\quad w_{n}\in Su_{n}, \\ x_{n+1}= \beta_{n} w_{n}+(1-\beta_{n})z_{n},\quad z_{n} \in Sy_{n}, \forall n\in\mathbb{N}, \end{cases}\displaystyle \end{aligned}$$

*where* $\{\alpha_{n}\}\subset(0,1)$, $\{\beta_{n}\}\subset(0,1)$, $\{r_{n}\}\subset(0,\infty)$, *and* $\gamma\in(0,\frac{1}{L})$ *such that* *L* *is the spectral radius of* $A^{*}A$ *and* $A^{*}$ *is the adjoint of* *A*. *Assume that the following conditions hold*:

1. $0<\liminf_{n\rightarrow\infty} \beta_{n}\leq\limsup_{n\rightarrow\infty}\beta_{n}<1$;
2. $0<\liminf_{n\rightarrow\infty} \alpha_{n}\leq\limsup_{n\rightarrow\infty}\alpha_{n}<1$;
3. $0<\liminf_{n\rightarrow\infty} r_{n}$.

*Then the sequence* $\{x_{n}\}$ *generated by* (3.3) *converges weakly to* $p\in\Theta$.

### Proof

First, we show that $A^{*}(I-T^{F_{2}}_{r_{n}})A$ is a $\frac{1}{L}$-inverse strongly monotone mapping. Since $T^{F_{2}}_{r_{n}}$ is firmly nonexpansive and $I-T^{F_{2}}_{r_{n}}$ is 1-inverse strongly monotone, we see that

$$\begin{aligned} \bigl\Vert A^{*} \bigl(I-T^{F_{2}}_{r_{n}} \bigr)Ax-A^{*} \bigl(I-T^{F_{2}}_{r_{n}} \bigr)Ay \bigr\Vert ^{2} &= \bigl\langle A^{*} \bigl(I-T^{F_{2}}_{r_{n}} \bigr) (Ax-Ay), A^{*} \bigl(I-T^{F_{2}}_{r_{n}} \bigr) (Ax-Ay) \bigr\rangle \\ &= \bigl\langle \bigl(I-T^{F_{2}}_{r_{n}} \bigr) (Ax-Ay), AA^{*} \bigl(I-T^{F_{2}}_{r_{n}} \bigr) (Ax-Ay) \bigr\rangle \\ &\leq L \bigl\langle \bigl(I-T^{F_{2}}_{r_{n}} \bigr) (Ax-Ay), \bigl(I-T^{F_{2}}_{r_{n}} \bigr) (Ax-Ay) \bigr\rangle \\ &= L \bigl\Vert \bigl(I-T^{F_{2}}_{r_{n}} \bigr) (Ax-Ay) \bigr\Vert ^{2} \\ &\leq L \bigl\langle Ax-Ay, \bigl(I-T^{F_{2}}_{r_{n}} \bigr) (Ax-Ay) \bigr\rangle \\ &= L \bigl\langle x-y, A^{*} \bigl(I-T^{F_{2}}_{r_{n}} \bigr)Ax-A^{*} \bigl(I-T^{F_{2}}_{r_{n}} \bigr)Ay \bigr\rangle \end{aligned}$$

for all $x,y \in H_{1}$. This implies that $A^{*}(I-T^{F_{2}}_{r_{n}})A$ is a $\frac{1}{L}$-inverse strongly monotone mapping. Since $\gamma\in (0,\frac{1}{L})$, it follows that $I-\gamma A^{*}(I-T^{F_{2}}_{r_{n}})A$ is a nonexpansive mapping.

Now, we divide the proof into five steps as follows:

*Step* 1. Show that $\{x_{n}\}$ is bounded.

Let $q\in\Theta$. Then we have $q=T^{F_{1}}_{r_{n}}q$ and $q=(I-\gamma A^{*}(I-T^{F_{2}}_{r_{n}})A)q$. By nonexpansiveness of $I-\gamma A^{*}(I-T^{F_{2}}_{r_{n}})A$, it implies that

3.4 $$\begin{aligned} \Vert u_{n}-q \Vert &= \bigl\Vert T^{F_{1}}_{r_{n}} \bigl(I-\gamma A^{*} \bigl(I-T^{F_{2}}_{r_{n}} \bigr)A \bigr)x_{n}-T^{F_{1}}_{r_{n}} \bigl(I-\gamma A^{*} \bigl(I-T^{F_{2}}_{r_{n}} \bigr)A \bigr)q \bigr\Vert \\ &\leq \bigl\Vert \bigl(I-\gamma A^{*} \bigl(I-T^{F_{2}}_{r_{n}} \bigr)A \bigr)x_{n}- \bigl(I-\gamma A^{*} \bigl(I-T^{F_{2}}_{r_{n}} \bigr)A \bigr)q \bigr\Vert \\ &\leq \Vert x_{n}-q \Vert . \end{aligned}$$

This implies that

3.5 $$ \Vert w_{n}-q \Vert =\operatorname{dist}(w_{n},Sq) \leq H(Su_{n},Sq)\leq \Vert u_{n}-q \Vert \leq \Vert x_{n}-q \Vert , $$

and so

3.6 $$\begin{aligned} \Vert y_{n}-q \Vert &= \bigl\Vert \alpha_{n} x_{n}+(1-\alpha_{n})w_{n}-q \bigr\Vert \\ &\leq\alpha_{n} \Vert x_{n}-q \Vert +(1- \alpha_{n}) \Vert w_{n}-q \Vert \\ &= \Vert x_{n}-q \Vert . \end{aligned}$$

It follows that

3.7 $$ \Vert z_{n}-q \Vert =\operatorname{dist}(z_{n},Sq) \leq H(Sy_{n},Sq)\leq \Vert y_{n}-q \Vert \leq \Vert x_{n}-q \Vert . $$

By (3.5) and (3.7), we have

3.8 $$\begin{aligned} \Vert x_{n+1}-q \Vert &= \bigl\Vert \beta_{n} w_{n}+(1-\beta_{n})z_{n}-q \bigr\Vert \\ &\leq \beta_{n} \Vert w_{n}-q \Vert +(1- \beta_{n}) \Vert z_{n}-q \Vert \\ &= \Vert x_{n}-q \Vert . \end{aligned}$$

This implies that $\{ \Vert x_{n}-q \Vert \}$ is decreasing and bounded below, thus $\lim_{n\longrightarrow \infty} \Vert x_{n}-q \Vert $ exists for all $q\in\Theta$.

*Step* 2. Show that $\lim_{n\rightarrow \infty} \Vert w_{n}-z_{n} \Vert =0$.

From Lemma 2.1(3), (3.5), (3.7), and $Sq=\{q\}$, we have

3.9 $$\begin{aligned} \Vert x_{n+1}-q \Vert ^{2}&= \bigl\Vert \beta_{n} w_{n}+(1-\beta_{n})z_{n}-q \bigr\Vert ^{2} \\ &\leq\beta_{n} \Vert w_{n}-q \Vert ^{2}+(1- \beta_{n}) \Vert z_{n}-q \Vert ^{2}- \beta_{n}(1-\beta _{n}) \Vert w_{n}-z_{n} \Vert ^{2} \\ &\leq \Vert x_{n}-q \Vert ^{2}-\beta_{n}(1- \beta_{n}) \Vert w_{n}-z_{n} \Vert ^{2}. \end{aligned}$$

This implies that

$$ \beta_{n}(1-\beta_{n}) \Vert w_{n}-z_{n} \Vert ^{2}\leq \Vert x_{n}-q \Vert ^{2}- \Vert x_{n+1}-q \Vert ^{2}. $$

From Condition (C1) and $\lim_{n\rightarrow\infty} \Vert x_{n}-q \Vert $ exists, we have

3.10 $$ \lim_{n\rightarrow\infty} \Vert w_{n}-z_{n} \Vert =0. $$

*Step* 3. Show that $\lim_{n\rightarrow\infty} \Vert u_{n}-x_{n} \Vert =0$ and $\lim_{n\rightarrow\infty} \Vert w_{n}-u_{n} \Vert =0$.

For $q\in\Theta$, we see that

$$\begin{aligned} \Vert u_{n}-q \Vert ^{2}={}& \bigl\Vert T^{F_{1}}_{r_{n}} \bigl(I-\gamma A^{*} \bigl(I-T^{F_{2}}_{r_{n}} \bigr)A \bigr)x_{n}-T^{F_{1}}_{r_{n}}q \bigr\Vert ^{2} \\ \leq{}& \bigl\Vert \bigl(I-\gamma A^{*} \bigl(I-T^{F_{2}}_{r_{n}} \bigr)A \bigr)x_{n}-q \bigr\Vert ^{2} \\ \leq{}& \Vert x_{n}-q \Vert ^{2}+\gamma^{2} \bigl\Vert A^{*} \bigl(I-T^{F_{2}}_{r_{n}} \bigr)Ax_{n} \bigr\Vert ^{2}+2\gamma \bigl\langle q-x_{n}, A^{*} \bigl(I-T^{F_{2}}_{r_{n}} \bigr)Ax_{n} \bigr\rangle \\ \leq {}&\Vert x_{n}-q \Vert ^{2}+\gamma^{2} \bigl\langle Ax_{n}-T^{F_{2}}_{r_{n}}Ax_{n}, AA^{*} \bigl(I-T^{F_{2}}_{r_{n}} \bigr)Ax_{n} \bigr\rangle \\ & {}+ 2\gamma \bigl\langle A(q-x_{n}), Ax_{n}-T^{F_{2}}_{r_{n}}Ax_{n} \bigr\rangle \\ \leq{}& \Vert x_{n}-q \Vert ^{2}+L\gamma^{2} \bigl\langle Ax_{n}-T^{F_{2}}_{r_{n}}Ax_{n}, Ax_{n}-T^{F_{2}}_{r_{n}}Ax_{n} \bigr\rangle \\ &{} + 2\gamma \bigl\langle A(q-x_{n})+ \bigl(Ax_{n}-T^{F_{2}}_{r_{n}}Ax_{n} \bigr) \\ &{}- \bigl(Ax_{n}-T^{F_{2}}_{r_{n}}Ax_{n} \bigr), Ax_{n}-T^{F_{2}}_{r_{n}}Ax_{n} \bigr\rangle \\ \leq{}& \Vert x_{n}-q \Vert ^{2}+L\gamma^{2} \bigl\Vert Ax_{n}-T^{F_{2}}_{r_{n}}Ax_{n} \bigr\Vert ^{2} \\ &{} + 2\gamma \bigl( \bigl\langle Ap-T^{F_{2}}_{r_{n}}Ax_{n}, Ax_{n}-T^{F_{2}}_{r_{n}}Ax_{n} \bigr\rangle - \bigl\Vert Ax_{n}-T^{F_{2}}_{r_{n}}Ax_{n} \bigr\Vert ^{2} \bigr) \\ \leq{}& \Vert x_{n}-q \Vert ^{2}+L\gamma^{2} \bigl\Vert Ax_{n}-T^{F_{2}}_{r_{n}}Ax_{n} \bigr\Vert ^{2} \\ &{} + 2\gamma \biggl(\frac{1}{2} \bigl\Vert Ax_{n}-T^{F_{2}}_{r_{n}}Ax_{n} \bigr\Vert ^{2}- \bigl\Vert Ax_{n}-T^{F_{2}}_{r_{n}}Ax_{n} \bigr\Vert ^{2} \biggr) \\ ={}& \Vert x_{n}-q \Vert ^{2}+\gamma(L\gamma-1) \bigl\Vert Ax_{n}-T^{F_{2}}_{r_{n}}Ax_{n} \bigr\Vert ^{2}. \end{aligned}$$

Thus, by (3.5) and (3.7), we have

3.11 $$\begin{aligned} \Vert x_{n+1}-q \Vert ^{2}&\leq \beta_{n} \Vert w_{n}-q \Vert ^{2}+(1- \beta_{n}) \Vert z_{n}-q \Vert ^{2} \\ &\leq \beta_{n} \Vert u_{n}-q \Vert ^{2}+(1- \beta_{n}) \Vert x_{n}-q \Vert ^{2} \\ &\leq \beta_{n} \bigl( \Vert x_{n}-q \Vert ^{2}+\gamma(L\gamma-1) \bigl\Vert Ax_{n}-T^{F_{2}}_{r_{n}}Ax_{n} \bigr\Vert ^{2} \bigr)+(1-\beta_{n}) \Vert x_{n}-q \Vert ^{2} \\ &\leq \Vert x_{n}-q \Vert ^{2}+\gamma(L\gamma-1) \beta_{n} \bigl\Vert Ax_{n}-T^{F_{2}}_{r_{n}}Ax_{n} \bigr\Vert ^{2}. \end{aligned}$$

Therefore, we have

$$\begin{aligned} -\gamma(L\gamma-1)\beta_{n} \bigl\Vert Ax_{n}-T^{F_{2}}_{r_{n}}Ax_{n} \bigr\Vert ^{2}\leq \Vert x_{n}-q \Vert ^{2}- \Vert x_{n+1}-q \Vert ^{2}. \end{aligned}$$

Since $\gamma(L\gamma-1)<0$, it follows by Condition (C1) and the existence of $\lim_{n\rightarrow\infty} \Vert x_{n}-q \Vert $ that

3.12 $$ \lim_{n\rightarrow\infty} \bigl\Vert Ax_{n}-T^{F_{2}}_{r_{n}}Ax_{n} \bigr\Vert =0. $$

Since $T^{F_{1}}_{r_{n}}$ is firmly nonexpansive and $I-\gamma A^{*}(I-T^{F_{2}}_{r_{n}})A$ is nonexpansive, we have

$$\begin{aligned} \Vert u_{n}-q \Vert ^{2}={}& \bigl\Vert T^{F_{1}}_{r_{n}} \bigl(I-\gamma A^{*} \bigl(I-T^{F_{2}}_{r_{n}} \bigr)A \bigr)x_{n}-T^{F_{1}}_{r_{n}}q \bigr\Vert ^{2} \\ \leq{}& \bigl\langle T^{F_{1}}_{r_{n}} \bigl(I-\gamma A^{*} \bigl(I-T^{F_{2}}_{r_{n}} \bigr)A \bigr)x_{n}-T^{F_{1}}_{r_{n}}q, \bigl(I-\gamma A^{*} \bigl(I-T^{F_{2}}_{r_{n}} \bigr)A \bigr)x_{n}-q \bigr\rangle \\ ={} &\bigl\langle u_{n}-q, \bigl(I-\gamma A^{*} \bigl(I-T^{F_{2}}_{r_{n}} \bigr)A \bigr)x_{n}-q \bigr\rangle \\ ={} &\frac{1}{2} \bigl( \Vert u_{n}-q \Vert ^{2}+ \bigl\Vert \bigl(I-\gamma A^{*} \bigl(I-T^{F_{2}}_{r_{n}} \bigr)A \bigr)x_{n}-q \bigr\Vert ^{2} \\ &{}- \bigl\Vert u_{n}-x_{n} -\gamma A^{*} \bigl(I-T^{F_{2}}_{r_{n}} \bigr)Ax_{n} \bigr\Vert ^{2} \bigr) \\ \leq{}&\frac{1}{2} \bigl( \Vert u_{n}-q \Vert ^{2}+ \Vert x_{n}-q \Vert ^{2}- \bigl( \Vert u_{n}-x_{n} \Vert ^{2} +\gamma^{2} \bigl\Vert A^{*} \bigl(I-T^{F_{2}}_{r_{n}} \bigr)Ax_{n} \bigr\Vert ^{2} \\ &{} -2\gamma \bigl\langle u_{n}-x_{n},A^{*} \bigl(I-T^{F_{2}}_{r_{n}} \bigr)Ax_{n} \bigr\rangle \bigr) \bigr), \end{aligned}$$

which implies that

3.13 $$\begin{aligned} \Vert u_{n}-q \Vert ^{2} &\leq \Vert x_{n}-q \Vert ^{2}- \Vert u_{n}-x_{n} \Vert ^{2}+2\gamma \bigl\langle u_{n}-x_{n},A^{*} \bigl(I-T^{F_{2}}_{r_{n}} \bigr)Ax_{n} \bigr\rangle \\ &\leq \Vert x_{n}-q \Vert ^{2}- \Vert u_{n}-x_{n} \Vert ^{2}+2\gamma \Vert u_{n}-x_{n} \Vert \bigl\Vert A^{*} \bigl(I-T^{F_{2}}_{r_{n}} \bigr)Ax_{n} \bigr\Vert . \end{aligned}$$

This implies by (3.5) and (3.7) that

$$\begin{aligned} \Vert x_{n+1}-q \Vert ^{2} \leq{}& \beta_{n} \Vert w_{n}-q \Vert ^{2}+(1- \beta_{n}) \Vert z_{n}-q \Vert ^{2} \\ \leq{}& \beta_{n} \Vert u_{n}-q \Vert ^{2}+(1- \beta_{n}) \Vert x_{n}-q \Vert ^{2} \\ \leq{}& \beta_{n} \bigl( \Vert x_{n}-q \Vert ^{2}- \Vert u_{n}-x_{n} \Vert ^{2}+2 \gamma \Vert u_{n}-x_{n} \Vert \bigl\Vert A^{*} \bigl(I-T^{F_{2}}_{r_{n}} \bigr)Ax_{n} \bigr\Vert \bigr) \\ &{}+(1-\beta_{n}) \Vert x_{n}-q \Vert ^{2}. \end{aligned}$$

Therefore, we have

$$\begin{aligned} \beta_{n} \Vert u_{n}-x_{n} \Vert ^{2} &\leq \Vert x_{n}-q \Vert ^{2}- \Vert x_{n+1}-q \Vert ^{2}+2\gamma\beta_{n} \Vert u_{n}-x_{n} \Vert \bigl\Vert A^{*} \bigl(I-T^{F_{2}}_{r_{n}} \bigr)Ax_{n} \bigr\Vert \\ &\leq \Vert x_{n}-q \Vert ^{2}- \Vert x_{n+1}-q \Vert ^{2}+2\gamma\beta_{n}M \bigl\Vert A^{*} \bigl(I-T^{F_{2}}_{r_{n}} \bigr)Ax_{n} \bigr\Vert , \end{aligned}$$

where $M=\sup\{ \Vert u_{n}-x_{n} \Vert : n\in\mathbb{N}\}$. This implies by Condition (C1), (3.12), and the existence of $\lim_{n\rightarrow \infty} \Vert x_{n}-q \Vert $ that

3.14 $$ \lim_{n\rightarrow\infty} \Vert u_{n}-x_{n} \Vert =0. $$

From (3.5), (3.7), and the definition of $\{y_{n}\}$, we obtain

$$\begin{aligned} \Vert x_{n+1}-q \Vert ^{2} \leq{}& \beta_{n} \Vert w_{n}-q \Vert ^{2}+(1- \beta_{n}) \Vert z_{n}-q \Vert ^{2} \\ \leq{}& \beta_{n} \Vert x_{n}-q \Vert ^{2}+(1- \beta_{n}) \Vert y_{n}-q \Vert ^{2} \\ ={}& \beta_{n} \Vert x_{n}-q \Vert ^{2}+(1- \beta_{n}) \bigl(\alpha_{n} \Vert x_{n}-q \Vert ^{2}+(1-\alpha _{n}) \Vert w_{n}-q \Vert ^{2} \\ &{}-\alpha_{n}(1-\alpha_{n}) \Vert x_{n}-w_{n} \Vert ^{2} \bigr) \\ \leq{}& \beta_{n} \Vert x_{n}-q \Vert ^{2}+(1- \beta_{n}) \bigl( \Vert x_{n}-q \Vert ^{2}- \alpha_{n}(1-\alpha _{n}) \Vert x_{n}-w_{n} \Vert ^{2} \bigr) \\ ={}& \Vert x_{n}-q \Vert ^{2}-\alpha_{n}(1- \alpha_{n}) (1-\beta_{n}) \Vert x_{n}-w_{n} \Vert ^{2}. \end{aligned}$$

This implies that

$$\begin{aligned} \alpha_{n}(1-\alpha_{n}) (1- \beta_{n}) \Vert x_{n}-w_{n} \Vert ^{2} \leq \Vert x_{n}-q \Vert ^{2}- \Vert x_{n+1}-q \Vert ^{2}. \end{aligned}$$

From Conditions (C1), (C2), and the existence of $\lim_{n\rightarrow \infty} \Vert x_{n}-q \Vert $, we have

3.15 $$ \lim_{n\rightarrow\infty} \Vert w_{n}-x_{n} \Vert =0. $$

By (3.14) and (3.15), we get

3.16 $$ \Vert w_{n}-u_{n} \Vert \leq \Vert w_{n}-x_{n} \Vert + \Vert x_{n}-u_{n} \Vert \rightarrow 0\quad \text{as } n\rightarrow\infty. $$

*Step* 4. Show that $\omega_{w}(x_{n})\subset\Theta$, where $\omega_{w}(x_{n})=\{x\in H_{1}:x_{n_{i}}\rightharpoonup x,\{x_{n_{i}}\}\subset\{x_{n}\}\}$. Since $\{x_{n}\}$ is bounded and $H_{1}$ is reflexive, $\omega_{w}(x_{n})$ is nonempty. Let $p\in \omega_{w}(x_{n})$ be an arbitrary element. Then there exists a subsequence $\{x_{n_{i}}\}\subset\{x_{n}\}$ converging weakly to *p*. From (3.14), it implies that $u_{n_{i}}\rightharpoonup p$ as $i\rightarrow\infty$. By (3.16) and Lemma 2.3, we have $p\in F(S)$.

Next, we show that $p\in \mathit{MEP}(F_{1},\varphi)$. Since $u_{n}=T^{F_{1}}_{r_{n}}(I-\gamma A^{*}(I-T^{F_{2}}_{r_{n}})A)x_{n}$, we have

$$ F_{1}(u_{n},y)+\varphi(y)-\varphi(u_{n})+ \frac{1}{r_{n}} \bigl\langle y-u_{n},u_{n}-x_{n} - \gamma A^{*} \bigl(I-T^{F_{2}}_{r_{n}} \bigr)Ax_{n} \bigr\rangle \geq0,\quad \forall y\in C, $$

which implies that

$$ F_{1}(u_{n},y)+\varphi(y)-\varphi(u_{n})+ \frac{1}{r_{n}}\langle y-u_{n},u_{n}-x_{n} \rangle-\frac{1}{r_{n}} \bigl\langle y-u_{n}, \gamma A^{*} \bigl(I-T^{F_{2}}_{r_{n}} \bigr)Ax_{n} \bigr\rangle \geq0,\quad \forall y\in C. $$

From Assumption 2.4(A2), we have

$$\begin{aligned} &\varphi(y)-\varphi(u_{n})+\frac{1}{r_{n}}\langle y-u_{n},u_{n}-x_{n} \rangle-\frac{1}{r_{n}} \bigl\langle y-u_{n}, \gamma A^{*} \bigl(I-T^{F_{2}}_{r_{n}} \bigr)Ax_{n} \bigr\rangle \\ &\quad \geq -F_{1}(u_{n},y)\geq F_{1}(y,u_{n}),\quad \forall y\in C, \end{aligned}$$

and hence

$$\begin{aligned} &\varphi(y)-\varphi(u_{n_{i}})+\frac{1}{r_{n_{i}}}\langle y-u_{n_{i}},u_{n_{i}}-x_{n_{i}} \rangle-\frac{1}{r_{n_{i}}} \bigl\langle y-u_{n_{i}}, \gamma A^{*} \bigl(I-T^{F_{2}}_{r_{n_{i}}} \bigr)Ax_{n_{i}} \bigr\rangle \geq F_{1}(y,u_{n_{i}}),\\ &\quad\forall y\in C. \end{aligned}$$

This implies by $u_{n_{i}}\rightharpoonup p$, Condition (C3), (3.12), (3.14), Assumption 2.4(A2), and the proper lower semicontinuity of *φ* that

$$ F_{1}(y,p)+\varphi(p)-\varphi(y)\leq0,\quad \forall y\in C. $$

Put $y_{t}=ty+(1-t)p$ for all $t\in(0,1]$ and $y\in C$. Consequently, we get $y_{t}\in C$ and hence $F_{1}(y_{t},p)+\varphi(p)-\varphi(y_{t})\leq 0$. So, by Assumption 2.4(A1)-(A4), we have

$$\begin{aligned} 0 &= F_{1}(y_{t},y_{t})-\varphi(y_{t})+ \varphi(y_{t}) \\ &\leq tF_{1}(y_{t},y)+(1-t)F_{1}(y_{t},p)+t \varphi(y)+(1-t)\varphi(p)-\varphi (y_{t}) \\ &\leq t \bigl(F_{1}(y_{t},y)+\varphi(y)- \varphi(y_{t}) \bigr). \end{aligned}$$

Hence, we have

$$ F_{1}(y_{t},y)+\varphi(y)-\varphi(y_{t})\geq0,\quad \forall y\in C. $$

Letting $t\rightarrow0$, by Assumption 2.4(A3) and the proper lower semicontinuity of *φ*, we have

$$ F_{1}(p,y)+\varphi(y)-\varphi(p)\geq0, \quad\forall y\in C. $$

This implies that $p\in \mathit{MEP}(F_{1},\varphi)$.

Since *A* is a bounded linear operator, we have $Ax_{n_{i}}\rightharpoonup Ap$. Then it follows from (3.12) that

3.17 $$ T^{F_{2}}_{r_{n_{i}}}Ax_{n_{i}}\rightharpoonup Ap \quad\text{as }i\rightarrow \infty. $$

By the definition of $T^{F_{2}}_{r_{n_{i}}}Ax_{n_{i}}$, we have

$$ F_{2} \bigl(T^{F_{2}}_{r_{n_{i}}}Ax_{n_{i}},y \bigr)+ \phi(y)-\phi \bigl(T^{F_{2}}_{r_{n_{i}}}Ax_{n_{i}} \bigr) + \frac{1}{r_{n_{i}}} \bigl\langle y-T^{F_{2}}_{r_{n_{i}}}Ax_{n_{i}},T^{F_{2}}_{r_{n_{i}}}Ax_{n_{i}}-Ax_{n_{i}} \bigr\rangle \geq0 ,\quad \forall y\in Q. $$

Since $F_{2}$ is upper semicontinuous in the first argument, it implies by (3.17) that

$$ F_{2}(Ap,y)+\phi(y)-\phi(Ap)\geq0 ,\quad \forall y\in Q. $$

This shows that $Ap\in \mathit{MEP}(F_{2},\phi)$. Therefore, $p\in \mathit{SMEP}(F_{1},\varphi,F_{2},\phi)$ and hence $p\in\Theta$.

*Step* 5. Show that $\{x_{n}\}$ converges weakly to an element of Θ. It is sufficient to show that $\omega_{w}(x_{n})$ is a singleton set. Let $p,q\in\omega_{w}(x_{n})$ and $\{x_{n_{k}}\}$, $\{x_{n_{m}}\}$ be two subsequences of $\{x_{n}\}$ such that $x_{n_{k}}\rightharpoonup p$ and $x_{n_{m}}\rightharpoonup q$. From (3.14), we also have $u_{n_{k}}\rightharpoonup p$ and $u_{n_{m}}\rightharpoonup q$. By (3.16) and Lemma 2.3, we see that $p,q\in F(S)$. Applying Lemma 2.2, we obtain $p=q$. This completes the proof. □

If $\varphi= \phi=0$ in (3.1) and (3.2), then the split mixed equilibrium problem reduces to split equilibrium problem. So, the following result can be obtained from Theorem 3.2 immediately.

### Theorem 3.3

*Let* *C* *be a nonempty closed convex subset of a real Hilbert space* $H_{1}$ *and* *Q* *be a nonempty closed convex subset of a real Hilbert space* $H_{2}$. *Let* $A: H_{1}\rightarrow H_{2}$ *be a bounded linear operator and* $S:C\rightarrow K(C)$ *a* *λ*-*hybrid multivalued mapping*. *Let* $F_{1}:C\times C\rightarrow\mathbb{R}$, $F_{2}:Q\times Q\rightarrow\mathbb{R}$ *be bifunctions satisfying Assumption* 2.4, *and* $F_{2}$ *is upper semicontinuous in the first argument*. *Assume that* $\Theta=F(S)\cap \mathit{SEP}(F_{1},F_{2}) \neq\emptyset$ *and* $Sp=\{p\}$ *for all* $p\in F(S)$. *Let* $\{x_{n}\}$ *be a sequence generated by* $x_{1}\in C$ *and*

3.18 $$\begin{aligned} \textstyle\begin{cases} u_{n}=T^{F_{1}}_{r_{n}} (I-\gamma A^{*} (I-T^{F_{2}}_{r_{n}} )A )x_{n}, \\ y_{n}=\alpha_{n}x_{n}+(1-\alpha_{n})w_{n},\quad w_{n}\in Su_{n}, \\ x_{n+1}= \beta_{n} w_{n}+(1-\beta_{n})z_{n},\quad z_{n} \in Sy_{n}, \forall n\in\mathbb{N}, \end{cases}\displaystyle \end{aligned}$$

*where* $\{\alpha_{n}\}\subset(0,1)$, $\{\beta_{n}\}\subset(0,1)$, $\{r_{n}\}\subset(0,\infty)$, *and* $\gamma\in(0,\frac{1}{L})$ *such that* *L* *is the spectral radius of* $A^{*}A$ *and* $A^{*}$ *is the adjoint of* *A*. *Assume that the following conditions hold*:

1. $0<\liminf_{n\rightarrow\infty} \beta_{n}\leq\limsup_{n\rightarrow\infty}\beta_{n}<1$;
2. $0<\liminf_{n\rightarrow\infty} \alpha_{n}\leq\limsup_{n\rightarrow\infty}\alpha_{n}<1$;
3. $0<\liminf_{n\rightarrow\infty} r_{n}$.

*Then the sequence* $\{x_{n}\}$ *generated by* (3.18) *converges weakly to* $p\in\Theta$.

### Remark 3.4

(i) Theorems 3.2 and 3.3 extend the corresponding one of Suantai *et al.* [13] and Kazmi and Rizvi [10] to *λ*-hybrid multivalued mapping and to a split mixed equilibrium problem. In fact, we present a new iterative algorithm for finding a common element of the set of solutions of split mixed equilibrium problems and the set of fixed points of *λ*-hybrid multivalued mappings in a real Hilbert space.
(ii) It is well known that the class of *λ*-hybrid multivalued mappings contains the classes of nonexpansive multivalued mappings, nonspreading multivalued mappings. Thus, Theorems 3.2 and 3.3 can be applied to these classes of mappings.

We give an example to illustrate Theorem 3.2 as follows.

### Example 3.5

Let $H_{1}=\mathbb{R}$, $H_{2}=\mathbb{R}$, $C=[-3,0]$, and $Q=(-\infty, 0]$. Let $A:H_{1}\longrightarrow H_{2}$ defined by $Ax=\frac{x}{2}$ for each $x\in H_{1}$. Then $A^{*}y=\frac{y}{2}$ for each $y\in H_{2}$. So, $L=\frac{1}{2}$ is the spectral radius of $A^{*}A$. Define a multivalued mapping $S:C\longrightarrow K(C)$ by

$$\begin{aligned} Sx= \textstyle\begin{cases} [-\frac{ \vert x \vert }{ \vert x \vert +1},0 ], & \mbox{$x\in[-3,-2)$;} \\ \{0\}, & \mbox{$x\in[-2,0]$.} \end{cases}\displaystyle \end{aligned}$$

It easy to see that *S* is 1-hybrid multivalued mapping with $F(S)=\{0\}$ and $S(0)=\{0\}$. For each $x,y\in C$, define the bifunction $F_{1}:C\times C\longrightarrow \mathbb{R}$ by $F_{1}(x,y)=xy+y-x-x^{2}$ and define $\varphi(x) =0$ for each $x\in C$. For each $u,v\in Q$, define the bifunction $F_{2}:Q\times Q\longrightarrow \mathbb{R}$ by $F_{2}(u,v)=uv+10v-10u-u^{2}$ and define $\phi(u) =0$ for each $u\in Q$.

Choose $\alpha_{n}=\frac{n}{5n+1}$, $\beta_{n}=\frac{n}{9n+1}$, $r_{n}=\frac{n}{n+1}$, and $\gamma=\frac{1}{15}$. It is easy to check that $F_{1}$, $F_{2}$, $\{\alpha_{n}\}$, $\{\beta_{n}\}$, $\{r_{n}\}$ satisfy all conditions in Theorem 3.2.

For each $x\in C$, we compute $T^{F_{2}}_{r}Ax$. Find *z* such that

$$\begin{aligned} 0&\leq F_{2}(z,y)+\varphi(y)- \varphi(z)+\frac{1}{r}\langle y-z,z-Ax\rangle\\ & = zy+10y-10z-z^{2} + \frac{1}{r} \biggl\langle y-z,z-\frac{x}{2} \biggr\rangle \\ &= (z+10) (y-z) + \frac{1}{r}(y-z) \biggl(z-\frac{x}{2} \biggr) \\ &= (y-z) \biggl((z+10) + \frac{1}{r} \biggl(z-\frac{x}{2} \biggr) \biggr) \end{aligned}$$

for all $y\in Q$. Thus, by Lemma 2.5(2), it follows that $z=\frac{x-20r}{2(1+r)}$. That is, $T^{F_{2}}_{r}Ax=\frac{x-20r}{2(1+r)}$ for each $x\in C$. Furthermore, we get

$$\begin{aligned} \bigl(I-\gamma A^{*} \bigl(I-T^{F_{2}}_{r} \bigr)A \bigr)x &=x- \frac{1}{15} A^{*} \bigl(Ax-T^{F_{2}}_{r}Ax \bigr) \\ &=x-\frac{1}{15} A^{*} \biggl(\frac{x}{2}-\frac{x-20r}{2(1+r)} \biggr) \\ &=x-\frac{1}{15} \biggl(\frac{x}{4}-\frac{x-20r}{4(1+r)} \biggr) \\ &=x \biggl(1-\frac{\gamma}{60} \biggr)-\frac{\gamma(x-20r)}{60(1+r)}. \end{aligned}$$

Next, we find $u\in C$ such that $F_{1}(u,v)+\varphi(y)- \varphi(z)+\frac{1}{r}\langle v-u,u-s\rangle\geq0$ for all $v\in C$, where $s=(I-\gamma A^{*}(I-T^{F_{2}}_{r})A)x$. Note that

$$\begin{aligned} 0\leq F_{1}(u,v)+\varphi(y)- \varphi(z)+\frac{1}{r}\langle v-u,u-s\rangle&= uv+v-u-u^{2} + \frac{1}{r} \langle v-u,u-s \rangle \\ &= (u+1) (v-u) + \frac{1}{r}(v-u) (u-s) \\ &= (v-u) \biggl((u+1)+\frac{1}{r}(u-s) \biggr). \end{aligned}$$

Thus, by Lemma 2.5(2), it follows that

$$u=\frac{s-r}{1+r}=\frac{59x-60r}{60(1+r)} - \frac{x-20r}{60(1+r)^{2}}. $$

Then the algorithm (3.3) becomes

3.19 $$\begin{aligned} \textstyle\begin{cases} u_{n}=\frac{59x_{n}-60r_{n}}{60(1+r_{n})} - \frac{x_{n}-20r_{n}}{60(1+r_{n})^{2}},\quad r_{n}=\frac{n}{n+1}, \\ y_{n}=\frac{n}{5n+1} x_{n}+ (1-\frac{n}{5n+1} )w_{n}, \\ x_{n+1}= \frac{n}{9n+1} w_{n}+ (1- \frac{n}{9n+1} )z_{n},\quad \forall n\in\mathbb{N}, \end{cases}\displaystyle \end{aligned}$$

where

$$\begin{aligned} w_{n}= \textstyle\begin{cases} [-\frac{ \vert u_{n} \vert }{ \vert u_{n} \vert +1},0 ], & \mbox{$u_{n}\in[-3,-2)$;} \\ \{0\}, & \mbox{$u_{n}\in[-2,0]$,} \end{cases}\displaystyle \qquad z_{n}= \textstyle\begin{cases} [-\frac{ \vert y_{n} \vert }{ \vert y_{n} \vert +1},0 ], & \mbox{$y_{n}\in[-3,-2)$;} \\ \{0\}, & \mbox{$y_{n}\in[-2,0]$.} \end{cases}\displaystyle \end{aligned}$$

We choose $w_{n}=-\frac{ \vert u_{n} \vert }{ \vert u_{n} \vert +1}$ if $u_{n}\in[-3,-2)$ and $z_{n}=-\frac{ \vert y_{n} \vert }{ \vert y_{n} \vert +1}$ if $y_{n}\in[-3,-2)$. By using SciLab, we compute the iterates of (3.19) for the initial point $x_{1}=-3$. The numerical experiment’s results of our iteration for approximating the point 0 are given in Table 1.

**Table 1:** **Numerical results of Example** **3.5** **for the algorithm** (**3.19**)

***n***	$\boldsymbol {x_{n}}$	$\boldsymbol {\Vert x_{n}-x_{n-1} \Vert } $
1	−3.0000000*e* + 00	-
2	−6.8786127*e* − 02	2.9312139*e* + 00
3	0.0000000*e* + 00	6.8786127*e* − 02
4	0.0000000*e* + 00	0.0000000*e* + 00

## Conclusions

The results presented in this paper extend and generalize the work of Suantai *et al.* [13] and Kazmi and Rizvi [10]. The main aim of this paper is to propose an iterative algorithm to find an element for solving a class of split mixed equilibrium problems and fixed point problems for *λ*-hybrid multivalued mappings under weaker conditions. Some sufficient conditions for the weak convergence of such proposed algorithm are given. Also, in order to show the significance of the considered problem, some important applications are discussed.

## References

[1] Ceng LC, Yao JC (2008). A hybrid iterative scheme for mixed equilibrium problems and fixed point problems. J. Comput. Appl. Math..

[2] Blum E, Oettli W (1994). From optimization and variational inequalities to equilibrium problems. Math. Stud..

[3] Combettes PI, Hirstoaga SA (2005). Equilibrium programming in Hilbert spaces. J. Nonlinear Convex Anal..

[4] Flam SD, Antipin AS (1997). Equilibrium programming using proximal-like algorithm. Math. Program..

[5] Censor Y, Elfving T (1994). A multiprojection algorithm using Bregman projections in a product space. Numer. Algorithms.

[6] Censor Y, Bortfeld T, Martin B, Trofimov A (2006). A unified approach for inversion problems in intensity-modulated radiation therapy. Phys. Med. Biol..

[7] Censor Y, Elfving T, Kopf N, Bortfeld T (2005). The multiple-sets split feasibility problem and its applications for inverse problems. Inverse Probl..

[8] Censor Y, Motova A, Segal A (2007). Perturbed projections and subgradient projections for the multiple-sets split feasibility problem. J. Math. Anal. Appl..

[9] Chan T, Shen J (2005). Image Processing and Analysis: Variational, PDE, Wavelet, and Stochastic Methods.

[10] Kazmi KR, Rizvi SH (2013). Iterative approximation of a common solution of a split equilibrium problem, a variational inequality problem and a fixed point problem. J. Egypt. Math. Soc..

[11] Bnouhachem A (2013). Algorithms of common solutions for a variational inequality, a split equilibrium problem and a hierarchical fixed point problem. Fixed Point Theory Appl..

[12] Bnouhachem A (2014). Strong convergence algorithm for split equilibrium problems and hierarchical fixed point problems. Sci. World J..

[13] Suantai S, Cholamjiak P, Cho YJ, Cholamjiak W (2016). On solving split equilibrium problems and fixed point problems of nonspreading multi-valued mappings in Hilbert spaces. Fixed Point Theory Appl..

[14] Deepho J, Kumam W, Kumam P (2014). A new hybrid projection algorithm for solving the split generalized equilibrium problems and the system of variational inequality problems. J. Math. Model. Algorithms Oper. Res..

[15] Kumam W, Deepho J, Kumam P (2014). Hybrid extragradient method for finding a common solution of the split feasibility and system of equilibrium problems. Dyn. Contin. Discrete Impuls. Syst., Ser. B, Appl. Algorithms.

[16] Deepho J, Martinez-Moreno J, Kumam P (2016). A viscosity of Cesaro mean approximation method for split generalized equilibrium, variational inequality and fixed point problems. J. Nonlinear Sci. Appl..

[17] Opial Z (1967). Weak convergence of the sequence of successive approximation for nonexpansive mappings. Bull. Am. Math. Soc..

[18] Suantai S (2005). Weak and strong convergence criteria of Noor iterations for asymptotically nonexpansive mappings. J. Math. Anal. Appl..

[19] Iiduka H, Takahashi W (2006). Weak convergence theorem by Cesaro means for nonexpansive mappings and inverse-strongly monotone mappings. J. Nonlinear Convex Anal..

[20] Suantai S, Phuengrattana W (2015). Existence and convergence theorems for *λ*-hybrid mappings in Hilbert spaces. Dyn. Contin. Discrete Impuls. Syst. Ser. A, Math. Anal..

[21] Ma Z, Wang L, Chang SS, Duan W (2015). Convergence theorems for split equality mixed equilibrium problems with applications. Fixed Point Theory Appl..

